# Astragaloside IV attenuates inflammatory response mediated by NLRP‐3/calpain‐1 is involved in the development of pulmonary hypertension

**DOI:** 10.1111/jcmm.15671

**Published:** 2020-12-08

**Authors:** Yang Sun, Meili Lu, Tairan Sun, Hongxin Wang

**Affiliations:** ^1^ Key Laboratory of Cardiovascular and Cerebrovascular Drug Research of Liaoning Province Jinzhou Medical University Jinzhou China

**Keywords:** AS‐IV, calpain‐1, inflammation, monocrotaline, NLRP‐3

## Abstract

Inflammation eventually leads to pulmonary arterial hypertension (PAH). Astragaloside IV(AS‐IV) has a protective effect on pulmonary hypertension, but the specific protective mechanism has been unclear until now. Therefore, in this study, our aim was to investigate the mechanisms underlying the effects of AS‐IV on PAH. In vivo, male Sprague‐Dawley (SD) rats were injected intraperitoneally with monocrotaline (MCT, 60 mg/kg) and treated with AS‐IV (40 mg/kg, 80 mg/kg), MCC950 and MDL‐28170. In vitro, human pulmonary artery endothelial cells (HPAECs) were treated with monocrotaline pyrrole (MCTP, 60 μg/mL). The protein expression levels of NLRP‐3, caspase‐1, ASC, IL‐18, IL‐1β and calpain‐1 were measured in vivo and/or in vitro. The results showed that AS‐IV decreased the protein expression levels of NLRP‐3, caspase‐1, ASC, IL‐18, IL‐1β and calpain‐1 in vivo and/or vitro. In conclusion, in this study the results suggested that AS‐IV could inhibit monocrotaline‐induced pulmonary arterial hypertension via the NLRP‐3/calpain‐1 pathway.

## 1 INTRODUCTION

Pulmonary arterial hypertension (PAH) is a cardiovascular disease with high morbidity and mortality.^1^ Martinon et al proposed the concept of the 'inflammasome' for the first time in 2002.^2^ The NLRP‐3 inflammasome is a vital component of the innate immune system that mediates caspase‐1 activation, microbial infection and cellular damage via the pro‐inflammatory factor IL‐18/IL‐1β.^3^ Activation of the inflammasome involves a variety of mechanisms.^4^ In particular, calpain activation plays a critical role in the activation of NLRP‐3 inflammasome.^5^ Calpain is a family of calcium‐activated cysteine proteases located in the cytoplasm and mitochondria, which is activated under neutral (pH = 7) conditions. The calpain family has 15 members.^6^, ^7^ Studies have shown that calpain‐1 regulates inflammation by promoting the migration of inflammatory cells and the production of pro‐inflammatory cytokines. This indicates that calpain‐1 is closely related to inflammation.^8^

Astragaloside IV (AS‐IV) is a natural triterpenoid glycoside extracted from *Astragalus membranaceus*.^9^ Studies have shown that AS‐IV has a protective effect on pulmonary hypertension.^10^ AS‐IV inhibits NLRP‐3 inflammasome activation.^11^ However, the specific protective effect of AS‐IV on PAH that involves the NLRP‐3/calpain‐1 pathway remains unclear. Therefore, in this study, we investigated the protective effect of AS‐IV on PAH and the potential molecular intervention mechanisms involving the NLRP‐3/calpain‐1 pathway.

## 2 MATERIALS AND METHODS

### 2.1 Animal models and drug treatments

Male Sprague‐Dawley rats weighing 180‐200 g (Experimental Animal Center, Jinzhou Medical University, Jinzhou, China) were housed in cages with free access to food and water, and exposed to a 12‐hour light/dark cycle at a controlled temperature (25 ± 2°C). All animal treatment protocols used in this study were approved by the Animal Experimentation Ethics Committee of Jinzhou Medical University. After 1 week of pre‐adaptation, the rats were randomly divided into the following groups (all n = 10): (a) the control group; (b) the MCT group (60 mg/kg); (c) the MCT + MCC950 group (NLRP‐3 inhibitor, 10 mg/kg/d, intraperitoneal injection); (d) the MCT + MDL‐28170 group (calpain‐1 inhibitor, 20 mg/kg/d, intraperitoneal injection); (e) the MCT + AS‐IV40 mg/kg group; and (e) the MCT + AS‐IV 80 mg/kg. The model of PAH was produced by a single intraperitoneal injection of monocrotaline (60 mg/kg). AS‐IV was dissolved in 0.5% sodium carboxymethylcellulose (CMC‐Na). Rats in the AS‐IV group were administered 40 mg/kg and 80 mg/kg for 28 days by gavage after MCT administration. After 4 weeks of AS‐IV treatment, the rats were anesthetized with 20% urethane and then killed.

### 2.2 Immunohistochemical staining

After dewaxing and antigen repair, the slides were eliminated peroxidase activity and blocked non‐specific binding. The slides were incubated with primary antibodies for anti‐NLRP‐3 (1:100) at 4°C overnight, followed by incubation with an HRP‐conjugated secondary antibody. Then, the sections were stained with DAB and counterstained with haematoxylin. Image analysis was measured by Image‐Pro Plus software.

### 2.3 Immunofluorescence

The slides were permeabilized with 0.1% Triton X‐100 in PBS for 30 minutes and then incubated with 5% bovine serum albumin in PBS for 60 minutes. The slides were incubated with primary antibody against anti‐calpain‐1 (1:100) at 4°C overnight. Subsequently, the slides were incubated with the fluorescein isothiocyanate (FITC)‐conjugated goat anti‐rabbit secondary antibody and Hoechst 33258 staining solution. Image analysis was performed using Image‐Pro Plus software.

### 2.4 Enzyme‐linked immunosorbent assay (ELISA)

The contents of IL‐18 and IL‐1β in serum were detected according to the manufacturer's instructions for each kit.

### 2.5 Cell culture

Human pulmonary artery endothelial cells (HPAECs) were purchased from Tongpai Biotechnology Co., Ltd. The cells were incubated in endothelial cell medium (ECM) at 37°C in 5% carbon dioxide and 95% air, and 10% foetal bovine serum was added. HPAECs were induced by MCTP (60 μg/mL) for 24 hours, and then, HPAECs were treated with MCC950 (NLRP‐3 inhibitor; 10 μmol/L), MDL28170 (calpain‐1 inhibitor; 20 μmol/L) and AS‐IV (50 μmol/L and 100 μmol/L) for 30 minutes.

### 2.6 Western blotting

The collected lung tissue and HPAECs were homogenized in RIPA lysis buffer. The protein concentrations were measured using a BCA protein assay kit. The samples were separated by SDS‐PAGE (10%‐12% polyacrylamide gel) and transferred to a PVDF membrane, which was blocked with 1% BSA for 1.5 hours and then incubated with antibodies calpain‐1, NLRP‐3, caspase‐1, ASC, IL‐18, IL‐1β, GAPDH and β‐actin overnight at 4°C. The membrane was washed three times with TBST and then incubated at room temperature with an HRP‐conjugated secondary antibody for 2 hours. The results were analysed with ImageJ software.

### 2.7 Data analysis

The data are presented as the mean ± SD for normally distributed data and were analysed with SPSS 25.0. The data were processed with one‐way ANOVA. One‐way ANOVA with post hoc tests was performed for multiple‐group analysis. *P* < .05 was considered statistically significant.

## 3 RESULTS

### 3.1 AS‐IV inhibited the activation of the NLRP‐3/calpain‐1 pathway: In vivo and in vitro

**FIGURE 1:**
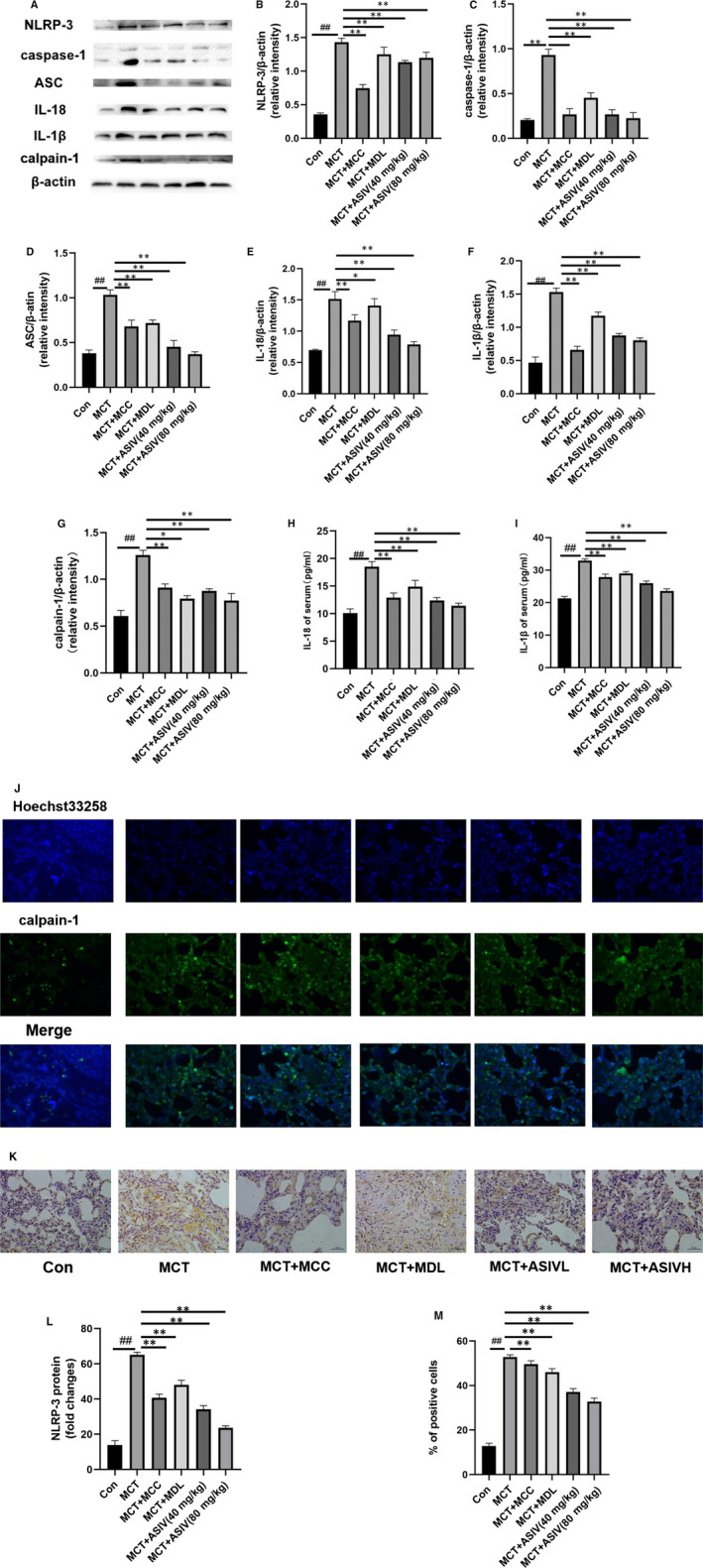
AS‐IV inhibited the activation of the NLRP‐3/calpain‐1 pathway in MCT‐induced rats. AS‐IV inhibited the release of NLRP‐3 inflammatory bodies and calpain‐1 in the pulmonary tissues of rats with pulmonary hypertension (A‐G). AS‐IV reduced the protein levels of IL‐18 and IL‐1β in the serum of rats with pulmonary hypertension (H, I). The expression of calpain‐1 in lung tissue was measured by immunofluorescence (J, M). The expression of NLRP‐3 in lung tissue was measured by immunohistochemistry (K, L). Original magnification, 400×. The data are presented as the mean ± SD (n = 3; ^##^ *P *< .01 vs Control group, *P < .05, **P < .01 vs MCT group) (AS‐IVL: AS‐IV 40 mg/kg, AS‐IVH: AS‐IV 80 mg/kg)

**FIGURE 2:**
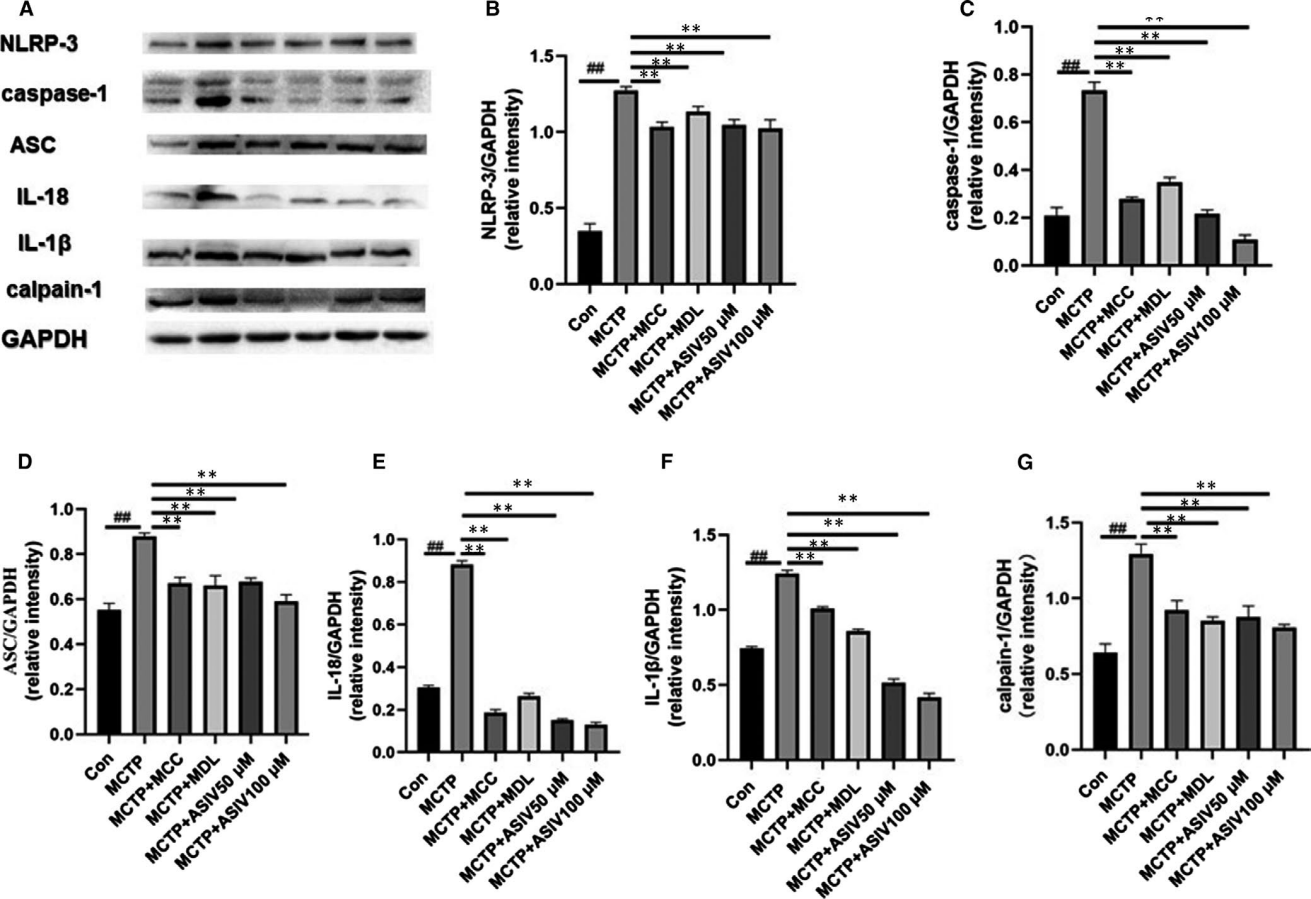
AS‐IV inhibited the activation of the NLRP‐3/calpain‐1 pathway in MCP‐induced HPAECs. The protein expression levels of NLRP‐3, caspase‐1, ASC, IL‐18, IL‐1β and calpain‐1 in HPAECs were measured by Western blotting (A‐G). The data are presented as the mean ± SD (n = 3; ^##^ *P *< .01 vs Control group, **P < .01 vs MCTP group)

To determine AS‐IV inhibited the release of inflammatory bodies. The protein expression levels of NLRP‐3, ASC, caspase‐1, IL‐18 and IL‐1β were detected by western blotting, immunohistochemistry,immunofluorescence and ELISA. The Western blotting results (Figure 1A‐G) showed that AS‐IV inhibited the release of NLRP‐3 inflammatory bodies and calpain‐1 in the pulmonary tissues of rats with pulmonary hypertension, and the inhibitory effect was similar to that of MCC950 and MDL28170. The ELISA results showed that AS‐IV reduced the levels of IL‐18 and IL‐1β in the serum of rats with pulmonary hypertension (Figure 1H,I), and the inhibitory effect was similar to that of MCC950 and MDL‐28170. The results of immunofluorescence (Figure 1J,M) and immunohistochemistry (Figure 1K,L) showed that AS‐IV inhibited the expression of calpain‐1 and NLRP‐3 in pulmonary tissue of rats with pulmonary hypertension, and the inhibitory effect was similar to that of MCC950 and MDL‐28170. The Western blotting results showed that AS‐IV inhibited the protein expression levels of NLRP‐3, caspase‐1, ASC, IL‐18, IL‐1β and calpain‐1 in HPAECs (Figure 2A‐G), and the inhibitory effect was similar to that of MCC950 and MDL28170.

## 4 DISCUSSION

The assembly of the NLRP‐3 inflammasome can lead to the activation of caspase‐1.^12^ Caspase‐1 is activated by proximity‐induced autocatalytic recruitment to the inflammasome. Active caspase‐1 cleaves the inflammatory factors pro‐IL‐18 and pro‐IL‐1β to generate mature IL‐18 and IL‐1β. Therefore, we tested whether AS‐IV could affect the NLRP‐3 activation pathway. Our data show that AS‐IV can inhibit the expression of NLRP‐3, caspase‐1, ASC, IL‐18 and IL‐1β in vivo and in vitro. Our study shows that NLRP‐3 is involved in regulating the expression of IL‐8 and IL‐1β in rats with pulmonary hypertension. In a recent report, Western blotting analysis confirmed that caspase‐1 activation and IL‐1β maturation in calpain‐1‐deficient cells were blocked.^13^ In addition, calpain inhibition or silencing attenuated myocardial ischaemia‐reperfusion injury in mice through the NLRP‐3/ASC/caspase‐1 axis.^14^ Therefore, we suggest that the inflammatory response mediated by NLRP‐3/calpain‐1 is involved in the development of pulmonary hypertension.

We used the NLRP‐3 inflammasome inhibitor (MCC950) and the calpain‐1 inhibitor (MDL‐28170) to verify that AS‐IV protected rats against pulmonary hypertension induced by MCT through the NLRP‐3/calpain‐1 signalling pathway. For the first time, we observed the improvement of the inflammatory response mediated by NLRP‐3/calpain‐1 as a breakthrough that could scientifically explain the prevention and treatment effects of AS‐IV on pulmonary hypertension. In addition, the pathogenesis of the underlying disease was mainly discussed from the perspective of the improvement of the effects on endothelial cells. However, the mechanism underlying the protective effect of AS‐IV on MCT‐induced pulmonary hypertension in rats mediated by the NLRP‐3/calpain‐1 signalling pathway is not clear. Therefore, we will generate calpain‐1 knockout mice to further clarify the specific mechanism.

## CONFLICT OF INTEREST

All authors claimed that there was no conflict of interest in the study.

## AUTHOR CONTRIBUTIONS

**Yang Sun:** Data curation (equal); methodology (equal); software (equal); validation (equal); writing‐review and editing (equal). **Meili Lu:** Data curation (equal); software (equal). **Tairan Sun :** Methodology (equal). **Hongxin Wang:** Funding acquisition (equal); project administration (equal).

## Data Availability

The data used to support the findings of this study are available from the corresponding author upon request.
